# Medical Items

**Published:** 1893-05

**Authors:** 


### *MEDICAL ITEMS.*

---

Dr. C. A. White, lately of Cartersville, has located in Atlanta.

---

Dr. R. C. Johnson, of Thomson, Ga., died of heart-disease, April 16th.

---

We have omitted from our issue this month some orignal communications and our regular New York letter, in order to give place to a full report of the Medical Association.

---

A bill has passed the Arkansas legislature providing for the suppression of prostitution in that State. Attempts at suppression probably result in more harm than good. Prostitution may be regulated, but not suppressed.

---

Dr. George J. Fisher, of Sing Sing, N. Y., died in March, of septicæmia, the result of a wound of the thumb received while doing an amputation. He was the author of several scholarly papers, relating principally to the history of medicine.

---

Dr. J. S. Dorsey Cullen died at his home in Richmond, Va., March 22d, in his sixty-first year. Dr. Cullen served through the war as Medical Director of General Longstreet's Corps. For the past twelve years he has been Professor of Surgery and Dean of the Faculty in the Medical College of Virginia, Richmond.

THE DeKalb County Medical Society of Alabama has lately decided that it will consider no application for license to practice unless the applicant gives satisfactory evidence of having had reasonable preparatory education. This will be severe on the proprietary college, which requires no entrance examination or the equivalent—but it will be just.

---

THE Legislature of Pennsylvania has passed an act establishing a Medical Council and three Boards of Medical Examiners. Members of the boards are appointed by the Governor from lists furnished by the several medical societies, and each board will examine the candidates of its own school. Applicants must have studied medicine *for at least four years.* The different boards will be under the supervision of the Medical Council.

---

A GRADUATE of one of the leading Philadelphia medical colleges applied for place as resident physician at a New Jersey hospital. He was asked to write a prescription for a fifty per cent. emulsion of cod-liver oil. Here it is:

℞. Cod-liver oil, ℥i.

Tinchor of iron, ℨii.

Sulphat of quinia, ℨi.

Syrupy simplicis, q. s.

Ad Oss.

---

THE conference of State Boards of Health, held in New York last week, grappled with the sanitary problems presented by the United States, especially in view of the possible coming epidemic of cholera. The spirit of the meeting showed a general belief that we shall have the disease among us, and that all arrangements must be made to prevent or overcome it. Surgeon-General Wyman reported the quarantine service along the Gulf and Atlantic coasts to be uniformly good. After much discussion the proposition to place a portion of the inspection service between State lines under the United States Marine-Hospital Service failed of adoption, though there was division of opinion among the members upon this point. In case the epidemic appears, it was recommended that the railroads should furnish separate cars for the use of the sick or "suspects," and suitable accommodations for them at places *en route* designated by the State Board of Health. The details of the medical inspection of trains, the disinfection of baggage, etc., were also considered. Delegates were, perhaps, too prone to be over-confident as to the sanitary conditions and the mechanisms of prophylaxis and care, as regards their own States or cities, but they probably believed that too great fear in such matters is quite as bad as too great confidence.—*Medical News.*

---

### ELEVENTH INTERNATIONAL MEDICAL CONGRESS.

ROME, ITALY, SEPT. 24TH—OCT. 1ST, 1893.

---

NEW YORK, April 1, 1893.

CIRCULAR:

The North German Lloyd, 2 Bowling Green, N. Y., offers a reduction of 25 per cent. to the medical men going to and coming from the Eleventh International Medical Congress, on steamer Werra, which is to sail from New York on August 5th and September 9th, and on steamer Fulda, on August 19th. Both these steamers sail to Genoa. The same reduction will be made for the return trips in October and November, on the same steamers, and for the Company's Saturday (off Bremen, Sunday off Southampton) steamers.

The Hamburg-American Packet Co., 37 Broadway, N. Y., 125 La Salle street, Chicago, offers a reduction of 25 per cent., both out and return, for all its steamers during the year 1893.

The Compagnie Generale Transatlantique, 3 Bowling Green, N. Y., offers the rates which are allowed French officers, that is, $63.50 for an $80 accommodation, and $91.50 for a $120 accommodation.

A. JACOBI, 110 W. 34th Street, N. Y.,
Chairman American National Committee.

